# AUV Underwater Positioning Algorithm Based on Interactive Assistance of SINS and LBL

**DOI:** 10.3390/s16010042

**Published:** 2015-12-30

**Authors:** Tao Zhang, Liping Chen, Yao Li

**Affiliations:** 1 Key Laboratory of Micro-Inertial Instrument and Advanced Navigation Technology, Ministry of Education, Southeast University, Nanjing 210096, China; 220132597@seu.edu.cn (L.C.); liyao@seu.edu.cn (Y.L.); 2 School of Instrument Science and Engineering, Southeast University, Nanjing 210096, China

**Keywords:** LBL, TDOA, underwater positioning, information fusion

## Abstract

This paper studies an underwater positioning algorithm based on the interactive assistance of a strapdown inertial navigation system (SINS) and LBL, and this algorithm mainly includes an optimal correlation algorithm with aided tracking of an SINS/Doppler velocity log (DVL)/magnetic compass pilot (MCP), a three-dimensional TDOA positioning algorithm of Taylor series expansion and a multi-sensor information fusion algorithm. The final simulation results show that compared to traditional underwater positioning algorithms, this scheme can not only directly correct accumulative errors caused by a dead reckoning algorithm, but also solves the problem of ambiguous correlation peaks caused by multipath transmission of underwater acoustic signals. The proposed method can calibrate the accumulative error of the AUV position more directly and effectively, which prolongs the underwater operating duration of the AUV.

## 1. Introduction

An AUV (autonomous underwater vehicle) is applied to execute all kinds of underwater tasks, including ocean exploration, underwater mine clearance and collecting bathymetry data of ocean and rivers [1,2,3,4]. In order to guarantee that underwater tasks will be completed smoothly and accurate underwater measurement data will be acquired, the AUV is required to have long-term autonomous high-precision positioning and navigation abilities and invisibility [5]. In an underwater environment, electromagnetic wave signals have the characteristic of serious attenuation. In deep sea or under an ice surface, adopting GPS and other radio positioning means cannot achieve ideal positioning effects. In order to meet the navigation requirements, DVL (Doppler velocity log) and SINS (strapdown inertial navigation system) are often used to integrate navigation [6], and the position will be estimated by dead reckoning. However, when this means is used for positioning, positioning errors will accumulate as time goes on [7]. When the AUV is performing tasks in shallow sea, it can adopt the navigation mode of “submerge, water surface calibration, submerge” to launch positioning and navigation; in other words, the AUV relies on SINS/DVL to launch positioning and navigation when navigating under water. After the AUV has been submerged under water for a certain time, in order to calibrate the accumulative errors, the AUV must emerge from the water, and the SINS/GPS integrated navigation system must be used to do the calibration [8]. Adopting this scheme can reach the goal of calibrating accumulative errors, but the AUV must be required to travel to and fro between the underwater operation position and the water surface, which will not only influence the working efficiency and increase the energy consumption, but also expose the position of the AUV. Especially when the AUV is operating in deep sea or under an ice surface, this scheme will be more impractical. Hence, it is very important to study a method in which reliable assistance positioning can be conducted for a long time underwater. This paper suggests an interactive assistance positioning method that integrates an LBL (long base line) underwater acoustic positioning system and an SINS/DVL/MCP (magnetic compass pilot) integrated navigation system, and this is a new idea for solving the above problems.

An LBL underwater acoustic positioning system, usually consisting of a seabed transponder matrix and an interrogation responder with a base length of hundreds to thousands of meters [9,10], adopts distance information between the underwater objective and the seabed matrix element to solve the target position. It can provide accurate positioning of an underwater vehicle within a local area without accumulative errors. Hence, an LBL underwater acoustic positioning system is very applicable to an underwater AUV to launch assistance positioning. Among some of the research of predecessors, Liu, Y. puts forward an underwater AUV positioning and navigation algorithm, which adopts an LBL underwater acoustic positioning system, an ADCP (acoustic Doppler current profiler) and depthometer-assisting INS [11]. Miller, P.A. *et al.* puts forward a tight integrated system based on LBL/DVL/INS [3]. Cheng,W.H. proposes a modification method, which is based on the periodically-measured actual navigation distance and is associated with the TOA positioning algorithm [12]. Jakuba, M.V. *et al.* report results for LBL acoustic navigation during autonomous under-ice surveys near the seafloor and adaptation of the LBL concept for several typical operational situations, including navigation in proximity to the ship during vehicle recoveries [13]. Eustice, R.M. *et al.* report recent experimental results in the development and deployment of a synchronous-clock acoustic navigation system suitable for the simultaneous navigation of multiple underwater vehicles [14]. Chen, Y.M. *et al.* propose a near-real-time approach to underwater inertial navigation with LBL, which uses a ping-response protocol, resulting in asynchronous measurements [15]. Although these systems have reached a certain positioning effect, there are some deficiencies. Firstly, these systems do not explain how to solve the positioning difficulty brought by the multi-path transmission of the underwater acoustic signal. In addition, acoustic velocity is distributed unevenly with the change of underwater depth, and sound ray transmission is curved, which will result in big positioning errors; additionally, the above systems have not proposed any solution.

When solving the target position, the LBL underwater acoustic positioning system can adopt the TOA (time of arrival) positioning algorithm and the TDOA (time difference of arrival) positioning algorithm. The equation set formulated by the TOA positioning algorithm can be directly transformed into a simple linear system of equations with a simple solution. However, strict time synchronization between the hydrophones and the sound source is required to measure a relatively accurate TOA value. It is very hard to do so in reality. The TDOA positioning algorithm acquires the TDOA value by conducting a generalized cross-correlation calculation of the signal received from one hydrophone and another, and then makes the positioning calculation. This method does not have to assure synchronization of the sound source and hydrophones, and the communication between them is quite simple. Hence, it is often adopted in wireless positioning. As acoustic signals will finally be in a coherence stack at one hydrophone through different paths, there will be multiple correlation peaks with approximate amplitudes in the generalized cross-correlation results, thus forming a phenomenon of ambiguous correlation peaks. Then, An, L. *et al.* came up with an ambiguity-solving algorithm based on underwater acoustic propagation characteristics [16]. This method, by studying the distribution rule of cross-correlation peaks forming the multipath transmission of underwater signal channels, tracks stable correlation peaks, which can effectively correct the miscalculation of the TDOA value caused by the ambiguity of the correlation peaks. However, under the condition that distances between fake peaks and the main peak do not differ that much, it is still very hard to accurately track, and the tracking error will be enlarged and finally diverge. In addition, underwater acoustic propagation channels will change as the underwater environment changes, so will the distribution rule of correlation peaks: if the former tracking strategy is still adopted, there will also be errors. Hence, the adaption of this tracking algorithm is not that strong.

In this paper, we propose an underwater positioning method based on the interactive assistance of LBL/SINS/DVL for an AUV. This positioning system consists of SINS, DVL and a sound source installed on the AUV and an LBL underwater matrix located at the seabed. The hydrophones of LBL receive signals from the sound source and conduct cross-correlation calculation and acquire the TDOA value. We adopt the hyperbolic model to solve the position of the sound source (namely, the position of the AUV), correct accumulative errors of SINS/DVL, use resolving results of SINS/DVL to assist in solving ambiguous correlation peaks when LBL is launching underwater acoustic positioning, estimate the TDOA value and improve the solution accuracy of LBL positioning. This scheme can not only directly correct accumulative errors caused by the dead reckoning algorithm, but also solves the problem of ambiguous correlation peaks caused by multipath transmission of underwater acoustic signals. Therefore, it is quite applicable to underwater positioning and navigation of an AUV.

The structure of this paper is as follows: Firstly, we introduce the principle and structure of the underwater assistance positioning system and then introduce key technologies of the system, such as underwater acoustic propagation channel modeling, the calculation of time delay differences, the Taylor series expansion algorithm, the TDOA position solution method and the interactive assistance algorithm. Finally, we verify the effectiveness of the algorithm through a simulation experiment.

## 2. Principle and Structure of the System

### 2.1. Placement and Positioning of the Hydrophone

The underwater LBL system needs to use a seabed hydrophone to confirm the position of the vehicle. The calculated position coordinates are the ones corresponding to the seabed hydrophone matrix. Hence, the hydrophone fixed on the seabed should be positioned firstly, and then, its absolute geographic position should be calculated.

**Figure 1:**
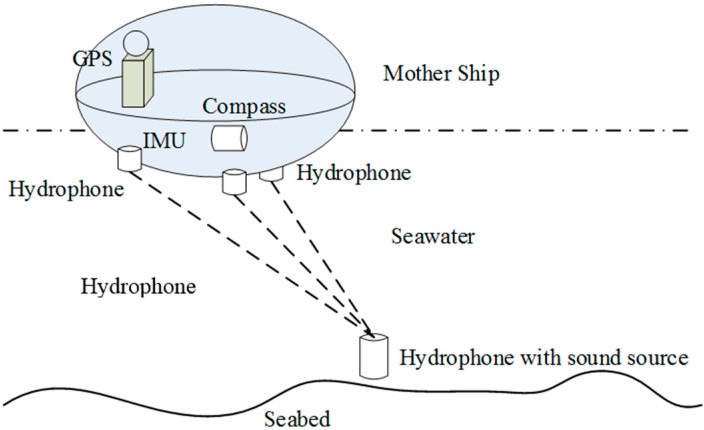
Positioning of the seabed hydrophone.

As shown in Figure 1, we install the hydrophone reception matrix at the bottom of mother ship (at least three hydrophones, usually more than three), which will receive signals from the hydrophone (with the sound source) underwater and then calculate the three-dimensional position coordinates of each underwater hydrophone corresponding to the hydrophone matrix at the bottom of the mother ship according to short base line positioning principles. The GPS, IMU and compass are installed on the mother ship to provide the accurate geographic position (longitude, latitude and depth) of the mother ship, as well as the attitude angle. We combine factors, such as this information of the mother ship and the installation errors, and calculate the absolute geographic position of each hydrophone under geodetic coordinates. The underwater AUV can adopt these hydrophones (their accurate positions are already acquired) to launch the local area positioning of itself.

### 2.2. LBL Underwater Positioning Model Based on TDOA

**Figure 2:**
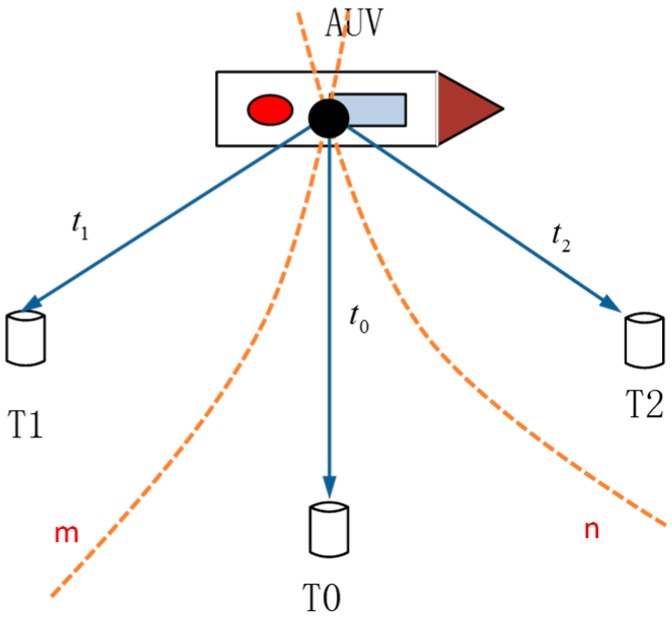
Schematic diagram of the TDOA positioning model.

TDOA positioning is a method that adopts delay inequality to perform the positioning. By measuring the time difference of a signal reaching different hydrophones, the distance difference between the signal source and different hydrophones can be acquired. As shown in Figure 2, suppose: hydrophones $T_{i}(i=0,1,2)$ located at three different positions; a sound source on the AUV sends a signal, and the transmission time of the signal reaching three hydrophones is $t_{i}(i=0,1,2)$; the sound velocity is a steady-state value (suppose it is c); then:

(1) $$R_{2}-R_{0}=c(t_{2}-t_{0})=c\Delta t_{20}$$

(2) $$R_{1}-R_{0}=c(t_{1}-t_{0})=c\Delta t_{10}$$

$R_{i}(i=0,1,2)$ represents the distance between the sound source and the hydrophone $T_{i}$. The above two equations respectively represent a hyperbolic curve n, which takes $T_{0}$ and $T_{2}$ as focal points, and hyperbolic curve m, which takes $T_{0}$ and $T_{1}$ as focal points; their point of intersection is the position of the sound source. As a certain error exists in the measured distance difference, there may be a condition with no solution. Hence, in view of this condition of multiple hydrophones placed on the seabed, redundant information is usually used to acquire the position closest to the actual position.

### 2.3. Working Process of the System

**Figure 3:**
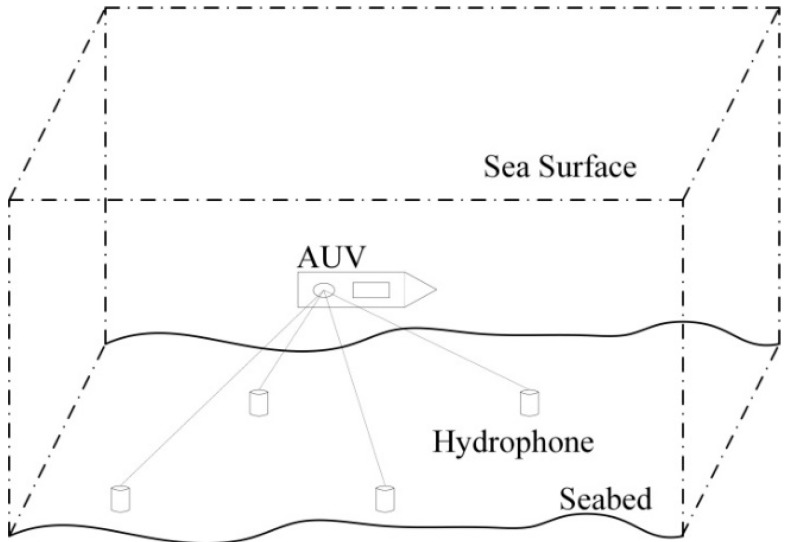
Schematic diagram of AUV underwater LBL positioning.

Figure 3 is a schematic diagram of AUV positioning based on LBL, with multiple fixed hydrophones on the seabed (there are four hydrophones in the diagram); a sound source fixed at the bottom of the AUV will send sound signals; first, accurate positioning of the hydrophones through sensors, like the GPS, IMU and compass, is performed, and the absolute geographic coordinates of each hydrophone are acquired, which is in preparation for solving the position of the sound source; then, a generalized correlation calculation of the sound source signals received by each hydrophone is done. As signals will be refracted and reflected during the transmission, multiple correlation peaks will be generated, resulting in the ambiguity of correlation peaks. Directed at this problem, this paper adopts SINS position assistance to estimate the time difference of sound source signals reaching each hydrophone, solves the distance difference according to the time difference and the equivalent transmission velocity of the signal and, finally, calculates the position of the sound source according to the hyperbolic positioning model. Hence, the interactive assistance positioning technology of SINS and LBL is an innovation point of this paper.

**Figure 4:**
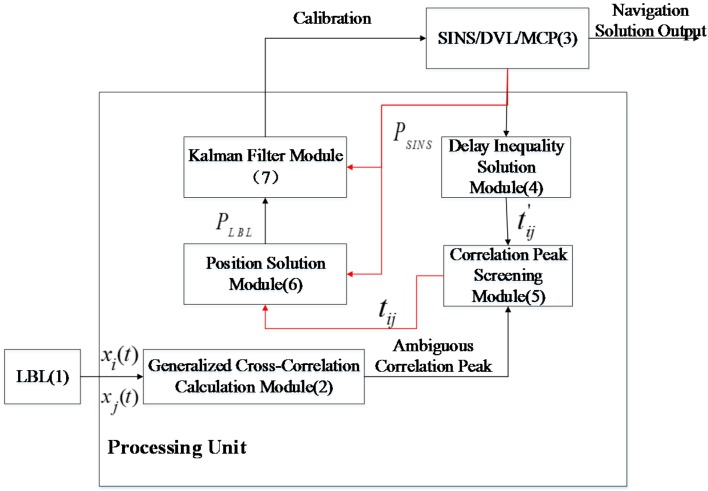
Operation principle block diagram of the system. SINS, strapdown inertial navigation system; DVL, Doppler velocity log; MCP, magnetic compass pilot.

Figure 4 is the operating block diagram of the system. The positioning system mainly consists of LBL, an SINS/DVL/MCP integrated system and a data processing unit. The solution will be made according to the sequence number in the box; firstly, the hydrophones in the LBL system receive signals (Box 1) from the sound source on the AUV and conduct a generalized correlation calculation of received signal ($x_{i}(t)$,$x_{j}(t)$) of hydrophone *i* and hydrophone *j*, and the calculation result is a group of ambiguous correlation peaks (Box 2). Then, the current AUV position information $P_{SINS}$ and the absolute position of the hydrophones (Box 3) according to the SINS/DVL/MCP integrated system are acquired, and the delay inequality $t_{ij}^{\prime }$ (Box 4) of the sound source signal reaching hydrophone *i* and hydrophone *j* is calculated. We adopt the correlation peak screening module again to screen the former acquired ambiguous correlation peaks and acquire the actual delay inequality $t_{ij}$ (Box 5), then we acquire the distance difference by combining the sound velocity correction algorithm, formulate a positioning solution equation, take $P_{SINS}$ as the initial iterative position, adopt the Taylor series expansion algorithm to solve AUV position $P_{LBL}$ (Box 6) in LBL positioning and, finally, input the difference value of $P_{LBL}$ and $P_{SINS}$ as the external observation information into a Kalman filter; velocity information provided by the DVL and heading information provided by the MCP are also taken as an observed quantity. Filtering results will correct the errors of SINS, and navigation results, such as the accurate position $P_{AUV}$, velocity and attitude of the AUV, will finally be acquired.

## 3. Principle of the Interactive Assistance Positioning Algorithm of SINS/DVL/MCP/LBL

This section introduces the realization principles of the algorithms in Figure 4, including generalized cross-correlation calculation of hydrophone receiving signals; SINS assists in seeking the ideal time differences and AUV position calculation based on TDOA.

### 3.1. Generalized Cross-Correlation Calculation of Hydrophone Receiving Signals

*x*(*t*) represents the sound source signal; suppose that the signal received by No. *i* hydrophone is:

(3) $$x_{i}(t)=\alpha_{i}x(t-\tau_{i})+n_{i}(t)$$

The signal received by No. *j* hydrophone is:

(4) $$x_{j}(t)=\alpha_{j}x(t-\tau_{j})+n_{j}(t)$$

$\alpha_{i}$ and $\alpha_{j}$ are attenuation coefficients of sound signals propagating underwater; $n_{i}(t)$ and $n_{j}(t)$ are non-correlative noise signals; $\tau_{i}$ and $\tau_{j}$ are propagation time.

The ross-correlation function of $x_{i}(t)$ and $x_{j}(t)$ is:

(5) $$R_{x_{i}x_{j}}(\tau )=E[x_{1}(t)x_{2}^{*}(t-\tau )]=\frac{1}{T-\tau }\int_{\tau }^{T}x_{i}(t)x_{j}(t-\tau )dt$$

$\tau =\tau_{j}-\tau_{i}$ represents TDOA; T is observation time. According to the characteristics of the correlation function, if the peak value of $R_{x_{i}x_{j}}(\tau )$ is found, then the corresponding τ is the right time difference.

### 3.2. Multi-Path Effect of Sound Signals Underwater

**Figure 5:**
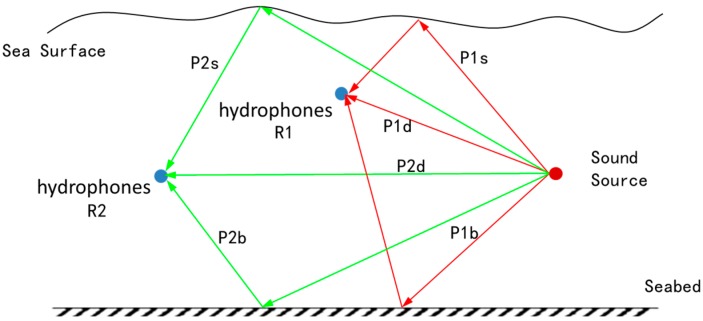
Simplified model of the underwater multi-path effect.

Figure 5 is a simplified multi-path underwater sound propagation model. Place a sound source and two hydrophones R1 and R2; simply consider nonstop path (P*i*d, *i*=1,2), sea surface reflection path (P*i*s, *i*=1,2) and seabed reflection path (P*i*b, *i*=1,2); set the sound source signal as *x*(*t*); then, the reception model of the hydrophone is as shown in Equation (6):

(6) $$\left\{ \begin{array}{l} x_{1}(t)=\alpha_{1D}x(t-\tau_{1D})+\alpha_{1S}x(t-\tau_{1S})+\alpha_{1B}x(t-\tau_{1B})+n_{1}(t)\\ x_{2}(t)=\alpha_{2D}x(t-\tau_{2D})+\alpha_{2S}x(t-\tau_{2S})+\alpha_{2B}x(t-\tau_{2B})+n_{2}(t) \end{array}\right.$$

**Figure 6:**
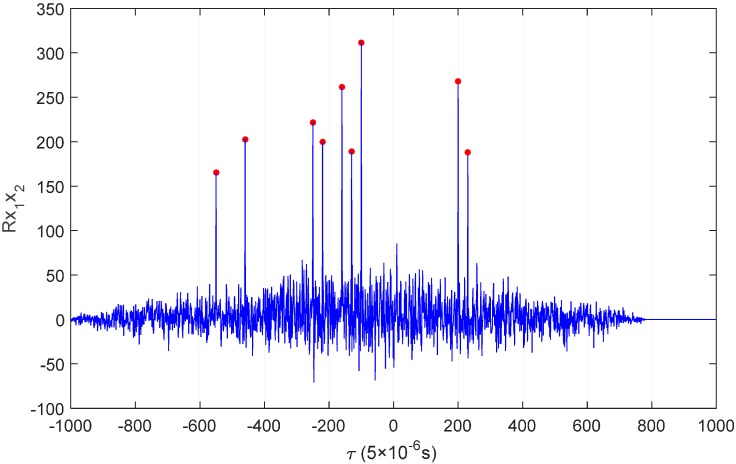
Ambiguity phenomenon of correlation peaks.

$\alpha_{iD}$, $\alpha_{iS}$ and $\alpha_{iB}$ are respectively attenuation coefficients of P*i*d path, P*i*s path and P*i*b path (*i* = 1,2). $\tau_{iD}$, $\tau_{iS}$ and $\tau_{iB}$ are respectively the propagation time of P*i*d path, P*i*s path and P*i*b path. Suppose that sound source signal x(t) is irrelevant to noise $n_{1}(t)$ and noise $n_{2}(t)$ and that $n_{1}(t)$ is irrelevant to noise $n_{2}(t)$, then the cross-correlation function of $x_{1}(t)$ and $x_{2}(t)$ is as shown in Equation (7). $R_{xx}(\tau )$ is a self-correlation function of x(t). It can be seen from Equation (7) that: peak values of the cross-correlation functions of $x_{1}(t)$ and $x_{2}(t)$ occur respectively on nine time delay of arrival points, such as $\tau_{1D}-\tau_{2D}$, $\tau_{1D}-\tau_{2S}$ and $\tau_{1D}-\tau_{2B}$ (nine peak values will occur when the nine points are unequal; if an equality situation among nine points exists, then there will be an overlapping phenomenon, and the number of peak values will reduce); the peak value will be decided by the corresponding attenuation coefficient. The specific effect is as shown in Figure 6. Under a practical situation, we only need to calculate the time difference of arrival of a nonstop path (main peak), so other peak values will interfere with confirming the main peak, which makes it impossible to accurately estimate the TDOA of signals.

(7) $$\begin{array}{ll} R_{x_{1}x_{2}}(\tau ) & =E[x_{1}(t)x_{2}^{*}(t-\tau )]\\ & =\alpha_{1D}\alpha_{2D}R_{xx}(\tau_{1D}-\tau_{2D}-\tau )\\ & +\alpha_{1D}\alpha_{2S}R_{xx}(\tau_{1D}-\tau_{2S}-\tau )\\ & +\alpha_{1D}\alpha_{2B}R_{xx}(\tau_{1D}-\tau_{2B}-\tau )\\ & +\alpha_{1S}\alpha_{2D}R_{xx}(\tau_{1S}-\tau_{2D}-\tau )\\ & +\alpha_{1S}\alpha_{2S}R_{xx}(\tau_{1S}-\tau_{2S}-\tau )\\ & +\alpha_{1S}\alpha_{2\text{B}}R_{xx}(\tau_{1S}-\tau_{2B}-\tau )\\ & +\alpha_{1B}\alpha_{2D}R_{xx}(\tau_{1B}-\tau_{2D}-\tau )\\ & +\alpha_{1B}\alpha_{2S}R_{xx}(\tau_{1B}-\tau_{2S}-\tau )\\ & +\alpha_{1B}\alpha_{2\text{B}}R_{xx}(\tau_{1B}-\tau_{2B}-\tau ) \end{array}$$

### 3.3. SINS/DVL Assistance in Seeking the Ideal Delay Inequality

Because of the multi-path effect, multiple correlation peaks will appear in the cross-correlation function, and these peaks differ a little in value; it is very difficult to judge which one corresponds to the most ideal time difference. In multi-path time delay estimation, the methods that can be adopted are usually the self-adaptation method, the generalized cross-correlation method, the auto-correlation method, the cepstrum method, *etc.* Although the self-adaptation method is of high estimation accuracy and a strong resolution ratio, its search scope is quite broad, which makes it hard to guarantee convergence and results in a large calculated quantity and poor instantaneity; besides, it has a certain requirement for the signal-to-noise ratio. The generalized cross-correlation method is of simple calculation, but the main correlation peak is not obvious; and there are certain deficiencies in its performance. Resolution ratios of the auto-correlation method and the cepstrum method are used for calculating the time delay, as they need a relatively larger signal bandwidth [17,18,19,20]. This paper opens up a new path by putting forward a method that selects the main correlation peak based on SINS/DVL positioning assistance after combining the advantages and disadvantages of the generalized cross-correlation method. This algorithm is simple and easy to realize; besides, it can estimate the time differences of multiple paths. Even with a low signal-to-noise ratio, it can effectively do the estimation; in the meantime, it can solve the problem of selecting the main correlation peak with high accuracy when correlation peaks are ambiguous. Now, the principle of the algorithm will be introduced in detail:

In the LBL underwater acoustics positioning system, set the position of No. *i* hydrophone as $P_{i}(x_{i},y_{i},z_{i})$ and the AUV position output by the SINS integrated system at time *k* as $P_{SINS}(x_{SINS}(k),y_{SINS}(k),z_{SINS}(k))$; adopt $P_{SINS}(x_{SINS}(k),y_{SINS}(k),z_{SINS}(k))$ to calculate the distance between the hydrophone and AUV at time k as:

(8) $$D_{i}(k)=\sqrt{{\left(x_{i}-x_{SINS}(k)\right)}^{2}+{\left(y_{i}-y_{SINS}(k)\right)}^{2}+{\left(z_{i}-z_{SINS}(k)\right)}^{2}}$$

The distance difference between two random hydrophones i and j and the AUV at time *k* is:

(9) $$D_{ij}(k)=D_{i}(k)-D_{j}(k)(i\neq j)$$

Then, the calculation of the time difference of the two hydrophones receiving signals is:

(10) $$\Delta t_{ij}^{\prime }(k)=\frac{D_{ij}(k)}{c_{ij}(k)}$$

$c_{ij}(k)$ is the equivalent sound velocity of signals corresponding to time difference $\Delta t_{ij}^{\prime }(k)$ at time *k*.

As at time *k* − 1, the surrounding environment of the AUV is not changed that much at time *k*, the change of the sound ray structure is little. Hence, the equivalent sound velocity of the last time cycle can be used as the current equivalent acoustic velocity; in other words, the velocity can be acquired by using the distance difference between hydrophones *i* and *j* and the sound source at time *k* − 1 to divide the time difference. The specific calculation is as follows:

At time *k* − 1, the corrected position of AUV by LBL is $P_{LBL/SINS}(x_{LBL/SINS}(k-1),y_{LBL/SINS}(k-1),z_{LBL/SINS}(k-1))$, and the distance of hydrophone *i* and the sound source is:

(11) $$R_{i}(k-1)=\sqrt{{\left(x_{i}-x_{LBL/SINS}(k-1)\right)}^{2}+{\left(y_{i}-y_{LBL/SINS}(k-1)\right)}^{2}+{\left(z_{i}-z_{LBL/SINS}(k-1)\right)}^{2}}$$

The distance difference between hydrophones i and j and the sound source is:

(12) $$\Delta R_{ij}(k-1)=R_{i}(k-1)-R_{j}(k-1)$$

If the time difference of hydrophones *i* and *j* (which have been screened out) receiving the signal at time $t_{k-1}$ is $\Delta t_{ij(k-1)}$, then the current equivalent acoustic velocity is:

(13) $$c_{ij}(k)=\frac{\Delta R_{ij}(k-1)}{\Delta t_{ij}(k-1)}$$

Substitute the computed results of Equations (9) and (13) into Equation (10), and time difference $\Delta t_{ij}^{\prime }(k)$ can be calculated. Seek the peak value that is the most proximate to $\Delta t_{ij}^{\prime }(k)$ among a group of ambiguous correlation peaks in Equation (7), and take the time difference corresponding to this peak value as the more accurate time difference $\Delta t_{ij}(k)$.

### 3.4. TDOA Three-Dimensional Positioning Algorithm Based on SINS/DVL Assistance

#### Three-Dimensional Positioning Algorithm Base on Taylor Series Expansion

For the moment, there are many algorithms that use TDOA measured to perform positioning, such as Chan’s algorithm, the Taylor algorithm, the Friedlander algorithm, *etc*. Chan’s algorithm has strict requirements for the measurement accuracy of the time difference and is more applicable to a line-of-sight transmission channel environment. Under a non-line-of-sight transmission condition, as for the measurement errors of the time difference of the signal arrival, the positioning errors of the algorithm will be large and will be easily influenced by the effects of reflection, scattering and refraction. What the Friedlander algorithm acquires is only the second-best solution. The Taylor algorithm has no special requirements for the statistic property of measuring errors or *a priori* information, and it can provide a higher positioning degree on a certain Gaussian noise level. However, this algorithm is an iterative one without a final expression solution, and algorithmic convergence needs to be guaranteed by an initial position that is not far from the actual position [21,22]. Hence, this paper suggests the TDOA positioning algorithm based on SINS/DVL assistance, and the algorithm takes the positioning results of SINS/DVL as the iterative initial value of the Taylor algorithm, which not only guarantees algorithmic convergence, but also reduces the iterations. It is very applicable to underwater positioning.

This algorithm will be introduced in detail as follows:

Suppose that there are *n* hydrophones in the matrix, then formulate (*n* – 1) equations according to the hyperbolic positioning model:

(14) $$R_{i1}=R_{i}-R_{1}=c_{i1}\Delta t_{i1}(i=2,3,4,\ldots ,n)$$

$R_{i1}$ is the function of $x,y,z,x_{i},y_{i},z_{i}$; (x,y,z) represents the position of the AUV; $\left(x_{i},y_{i},z_{i}\right)$ represents the position of No. *i* hydrophone; then, it can be expressed as $f_{i}(x,y,z,x_{i},y_{i},z_{i})=R_{i}-R_{1}$; set it as the objective function. Suppose that the measured value of objective function $f_{i}(*)$ (namely $f_{i}(x,y,z,x_{i},y_{i},z_{i})$) is $m_{i}=c_{i1}\Delta t_{i1}$, and the actual value is $u_{i}$, $u_{i}=m_{i}-e_{i}$, $e_{i}$ is the measuring error. Adopt initial value $\left(\hat{x},\hat{y},\hat{z}\right)$, and they meet $x=\hat{x}+\Delta x$, $y=\hat{y}+\Delta y$, $z=\hat{z}+\Delta z$; then, expand objective function $f_{i}(*)$ in $\left(\hat{x},\hat{y},\hat{z}\right)$ according to the Taylor series as the following Equation (15):

(15) $$\begin{array}{l} f_{i}(x,y,z,x_{i},y_{i},z_{i})=f_{i}(\hat{x},\hat{y},\hat{z},x_{i},y_{i},z_{i})+(\Delta x\frac{\partial }{\partial x}+\Delta y\frac{\partial }{\partial y}+\Delta z\frac{\partial }{\partial z})f_{i}(\hat{x},\hat{y},\hat{z},x_{i},y_{i},z_{i})\\ +\frac{1}{2!}{\left(\Delta x\frac{\partial }{\partial x}+\Delta y\frac{\partial }{\partial y}+\Delta z\frac{\partial }{\partial z}\right)}^{2}f_{i}(\hat{x},\hat{y},\hat{z},x_{i},y_{i},z_{i})+\cdots \\ +\frac{1}{n!}{\left(\Delta x\frac{\partial }{\partial x}+\Delta y\frac{\partial }{\partial y}+\Delta z\frac{\partial }{\partial z}\right)}^{n}f_{i}(\hat{x},\hat{y},\hat{z},x_{i},y_{i},z_{i})\\ +\frac{1}{(n+1)!}{\left(\Delta x\frac{\partial }{\partial x}+\Delta y\frac{\partial }{\partial y}+\Delta z\frac{\partial }{\partial z}\right)}^{n+1}f_{i}(\hat{x}+\xi \Delta x,\hat{y}+\xi \Delta y,\hat{z}+\xi \Delta z,x_{i},y_{i},z_{i}),(0<\xi <1) \end{array}$$

Ignore high-order terms above the quadratic term in the expanded section, then the above equation can be expressed as:

(16) $$f_{i}(x,y,z,x_{i},y_{i},z_{i})=u_{i}\approx f_{i}(\hat{x},\hat{y},\hat{z},x_{i},y_{i},z_{i})+(\Delta x\frac{\partial }{\partial x}+\Delta y\frac{\partial }{\partial y}+\Delta z\frac{\partial }{\partial z})f_{i}(\hat{x},\hat{y},\hat{z},x_{i},y_{i},z_{i})$$

It can be acquired according to the hyperbolic positioning model that:

(17) $$f_{i}(x,y,z,x_{i},y_{i},z_{i})=\sqrt{{\left(x-x_{i}\right)}^{2}+{\left(y-y_{i}\right)}^{2}+{\left(z-z_{i}\right)}^{2}}-\sqrt{{\left(x-x_{1}\right)}^{2}+{\left(y-y_{1}\right)}^{2}+{\left(z-z_{1}\right)}^{2}}$$

Expand Equation (17) according to Equation (16) and acquire:

(18) $$f_{i}(x,y,z,x_{i},y_{i},z_{i})\approx {\hat{R}}_{i}-{\hat{R}}_{1}+\Delta x(\frac{\hat{x}-x_{i}}{{\hat{R}}_{i}}-\frac{\hat{x}-x_{1}}{{\hat{R}}_{1}})+\Delta y(\frac{\hat{y}-y_{i}}{{\hat{R}}_{i}}-\frac{\hat{y}-y_{1}}{{\hat{R}}_{1}})+\Delta z(\frac{\hat{z}-z_{i}}{{\hat{R}}_{i}}-\frac{\hat{z}-z_{1}}{{\hat{R}}_{1}})$$

where:

(19) $${\hat{R}}_{1}=\sqrt{{\left(\hat{x}-x_{1}\right)}^{2}+{\left(\hat{y}-y_{1}\right)}^{2}+{\left(\hat{z}-z_{1}\right)}^{2}}$$

(20) $${\hat{R}}_{i}=\sqrt{{\left(\hat{x}-x_{i}\right)}^{2}+{\left(\hat{y}-y_{i}\right)}^{2}+{\left(\hat{z}-z_{i}\right)}^{2}}$$

Set ${\hat{f}}_{i}=f_{i}(\hat{x},\hat{y},\hat{z},x_{i},y_{i},z_{i})$, then:

(21) $$a_{i1}=\frac{\partial f_{i}(x,y,z,x_{i},y_{i},z_{i})}{\partial x}{|}_{\begin{array}{l} x=\hat{x}\\ y=\hat{y}\\ z=\hat{z} \end{array}}=\frac{\hat{x}-x_{i}}{{\hat{R}}_{i}}-\frac{\hat{x}-x_{1}}{{\hat{R}}_{1}}$$

(22) $$a_{i2}=\frac{\partial f_{i}(x,y,z,x_{i},y_{i},z_{i})}{\partial y}{|}_{\begin{array}{l} x=\hat{x}\\ y=\hat{y}\\ z=\hat{z} \end{array}}=\frac{\hat{y}-y_{i}}{{\hat{R}}_{i}}-\frac{\hat{y}-y_{1}}{{\hat{R}}_{1}}$$

(23) $$a_{i3}=\frac{\partial f_{i}(x,y,z,x_{i},y_{i},z_{i})}{\partial z}{|}_{\begin{array}{l} x=\hat{x}\\ y=\hat{y}\\ z=\hat{z} \end{array}}=\frac{\hat{z}-z_{i}}{{\hat{R}}_{i}}-\frac{\hat{z}-z_{1}}{{\hat{R}}_{1}}$$

and then:

(24) $${\hat{f}}_{i}+a_{i1}\Delta x+a_{i2}\Delta y+a_{i3}\Delta z\approx m_{i}-e_{i}$$

For the n matrix elements, there will be:

(25) ε≈h−Gδ

where:

(26) $$\varepsilon =\left[\begin{array}{c} e_{2}\\ e_{3}\\ \vdots \\ e_{n} \end{array}\right]$$

(27) $$h=\left[\begin{array}{c} m_{2}-{\hat{f}}_{2}\\ m_{3}-{\hat{f}}_{3}\\ \vdots \\ m_{n}-{\hat{f}}_{n} \end{array}\right]=\left[\begin{array}{c} c_{21}\Delta t_{21}-({\hat{R}}_{2}-{\hat{R}}_{1})\\ c_{31}\Delta t_{31}-({\hat{R}}_{3}-{\hat{R}}_{1})\\ \vdots \\ c_{n1}\Delta t_{n1}-({\hat{R}}_{n}-{\hat{R}}_{1}) \end{array}\right]$$

(28) $$G=\left[\begin{array}{ccc} a_{21} & a_{22} & a_{33}\\ a_{31} & a_{32} & a_{33}\\ \vdots & \vdots & \vdots \\ a_{n1} & a_{n2} & a_{n3} \end{array}\right]==\left[\begin{array}{ccc} \frac{\hat{x}-x_{2}}{{\hat{R}}_{2}}-\frac{\hat{x}-x_{1}}{{\hat{R}}_{1}} & \frac{\hat{y}-y_{2}}{{\hat{R}}_{2}}-\frac{\hat{y}-y_{1}}{{\hat{R}}_{1}} & \frac{\hat{z}-z_{2}}{{\hat{R}}_{2}}-\frac{\hat{z}-z_{1}}{{\hat{R}}_{1}}\\ \frac{\hat{x}-x_{3}}{{\hat{R}}_{3}}-\frac{\hat{x}-x_{1}}{{\hat{R}}_{1}} & \frac{\hat{y}-y_{3}}{{\hat{R}}_{3}}-\frac{\hat{y}-y_{1}}{{\hat{R}}_{1}} & \frac{\hat{z}-z_{3}}{{\hat{R}}_{3}}-\frac{\hat{z}-z_{1}}{{\hat{R}}_{1}}\\ \vdots & \vdots & \vdots \\ \frac{\hat{x}-x_{n}}{{\hat{R}}_{n}}-\frac{\hat{x}-x_{1}}{{\hat{R}}_{1}} & \frac{\hat{y}-y_{n}}{{\hat{R}}_{n}}-\frac{\hat{y}-y_{1}}{{\hat{R}}_{1}} & \frac{\hat{z}-z_{3}}{{\hat{R}}_{n}}-\frac{\hat{z}-z_{1}}{{\hat{R}}_{1}} \end{array}\right]$$

(29) $$\delta =\left[\begin{array}{c} \Delta x\\ \Delta y\\ \Delta z \end{array}\right]$$

Take δ as an unknown variable; suppose that Q is the covariance matrix of ε; then adopt the method of weighing least squares estimation; it can be acquired that:

(30) $$\left[\begin{array}{c} \Delta x\\ \Delta y\\ \Delta z \end{array}\right]=\delta ={\left[G^{T}Q^{-1}G\right]}^{-1}G^{T}Q^{-1}h$$

The calculation process of the Taylor algorithm can be concluded as follows:

(1) Select an initial value $\left(\hat{x},\hat{y},\hat{z}\right)$;
(2) Substitute $\left(\hat{x},\hat{y},\hat{z}\right)$ into Equations (20) and (28) to calculate G and ${\hat{R}}_{i}$;
(3) Substitute $c_{i1}$, $\Delta t_{21}$ and ${\hat{R}}_{i}$ into Equation (27) to calculate h;
(4) Substitute G, h and Q into Equation (30) and update δ; if $\sqrt{{\left(\Delta x\right)}^{2}+{\left(\Delta y\right)}^{2}+{\left(\Delta z\right)}^{2}}<\eta$, η is a very small threshold value, then the iteration ends; $\left(\hat{x},\hat{y},\hat{z}\right)$ is the final positioning result. Otherwise, update $\left(\hat{x},\hat{y},\hat{z}\right)$ according to Equation (31), and repeat $\left(\hat{x},\hat{y},\hat{z}\right)$ until the above conditions are met.
  (31) $$\left\{ \begin{array}{c} \hat{x}\leftarrow \hat{x}+\Delta x\\ \hat{y}\leftarrow \hat{y}+\Delta y\\ \hat{z}\leftarrow \hat{z}+\Delta z \end{array}\right.$$

**Figure 7:**
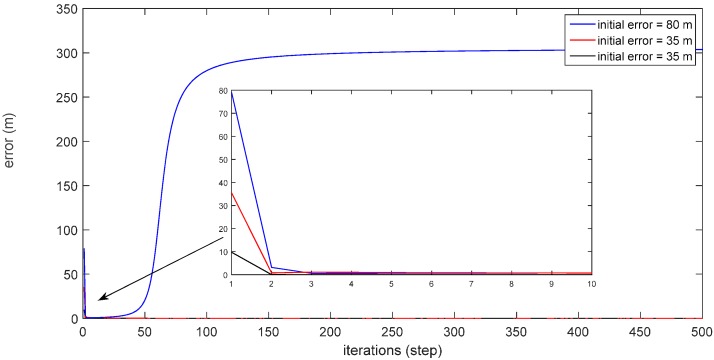
The iteration results with different initial position values.

The algorithm needs an estimated position value as an initial value to make iterations. The accuracy of the initial position value has great influence on the convergence of the algorithm. As shown in Figure 7, the convergence error threshold for the iterative algorithm is 10^−7^ m. When the initial position error is 80 m, the iterative result cannot converge. When the initial position error is 35 m, the iterative result converges, but the number of iterations is 468 steps. While the initial position error is set as 10 m, the number of iterations requires only 364 steps. Thus, the higher the accuracy of the initial position value, the faster the convergence rate. This paper selects the output position *P_SINS_* of SINS/DVL as the initial position value of the iteration, which can not only satisfy the convergence of the algorithm, but also greatly reduce the iteration steps.

### 3.5. SINS/DVL/MCP/LBL Integrated System Modeling

**Figure 8:**
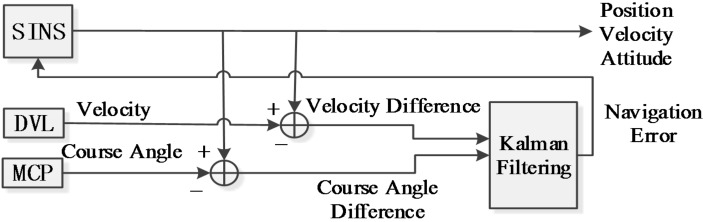
SINS/DVL/MCP integrated navigation system.

**Figure 9:**
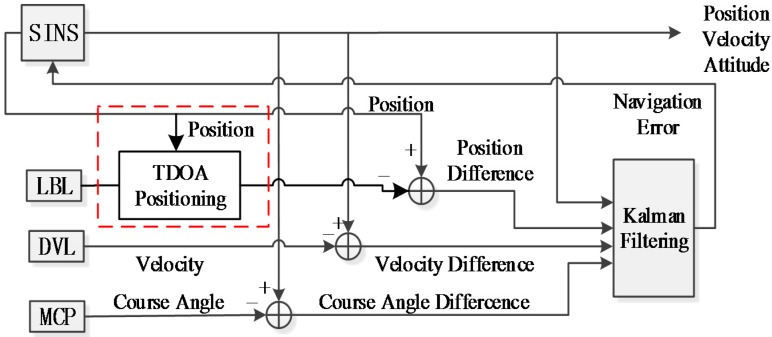
SINS/DVL/MCP/LBL integrated navigation system.

When the AUV does not enter the effective signal scope of LBL, adopt the SINS/DVL/MCP integrated navigation system as shown in Figure 8 to perform the navigation and positioning. When the AUV enters the effective signal scope of LBL, adopt the SINS/DVL/MCP/LBL integrated navigation system as shown in Figure 9 to perform the navigation and positioning.

The state equation of the integrated system is:

(32) $$\dot{X}=FX+GW$$

where **X** is the state variable, **F** is the state-transition matrix, **G** is the transition matrix of the process noise and **W** is systematic noise.

Select the velocity error, attitude error, accelerometer zero offset and gyroscopic drift as state vector **X**:

(33) $$X={\left[\begin{array}{ccccccccccccccc} \delta V_{E} & \delta V_{N} & \delta V_{U} & \phi_{E} & \phi_{N} & \phi_{U} & \delta L & \delta \lambda & \delta h & \nabla_{bx} & \nabla_{by} & \nabla_{bz} & \varepsilon_{bx} & \varepsilon_{by} & \varepsilon_{bz} \end{array}\right]}^{T}$$

$\delta V_{E},\delta V_{N},\delta V_{U}$ are respectively the velocity errors of the directions of east, north and the local vertical(up). $\phi_{E},\phi_{N},\phi_{U}$ are respectively the misalignment angles of the directions of east, north and the local vertical(up). δL,δλ,δh are respectively the errors of latitude, longitude and altitude. $\nabla_{bx},\nabla_{by},\nabla_{bz}$ are respectively biased errors of the three axial directions of the accelerator. $\varepsilon_{bx},\varepsilon_{by},\varepsilon_{bz}$ are respectively the drifts of the three axial directions of the gyroscopes. **F** can be confirmed by the SINS error equation.

The measuring equation of the integrated system is:

(34) $$V_{SINS}=\left[\begin{array}{c} V_{SINSE}\\ V_{SINSN}\\ V_{SINSU} \end{array}\right]$$

**Z** is the observation vector; $P_{SINS}=\left[\begin{array}{c} L_{SINS}\\ \lambda_{SINS}\\ h_{SINS} \end{array}\right]$ is the position information output of the SINS system; $P_{LBL}=\left[\begin{array}{c} L_{LBL}\\ \lambda_{LBL}\\ h_{LBL} \end{array}\right]$ is the position information output by the LBL system; $V_{SINS}=\left[\begin{array}{c} V_{SINSE}\\ \lambda_{SINSN}\\ h_{SINSU} \end{array}\right]$ is the velocity information output by the SINS system; $V_{DVL}=\left[\begin{array}{c} V_{DVLE}\\ V_{DVLN}\\ V_{DVLU} \end{array}\right]$ is the velocity information output by the DVL system; $\varphi_{U}$ is the heading information output by the SINS system; $\varphi_{MCP}$ is the heading information output by the MCP system; **V** is the observation noise vector; and **H** is the measurement matrix, which satisfies:

(35) $$H=\left[\begin{array}{ccccccccccccccc} 1 & 0 & 0 & 0 & 0 & 0 & 0 & 1 & 0 & 0 & 0 & 0 & 0 & 0 & 0\\ 0 & 1 & 0 & 0 & 0 & 0 & 0 & 0 & 1 & 0 & 0 & 0 & 0 & 0 & 0\\ 0 & 0 & 1 & 0 & 0 & 1 & 0 & 0 & 0 & 1 & 0 & 0 & 0 & 0 & 0 \end{array}\right]$$

## 4. Simulation and Experiment

### 4.1. Underwater Sound Signal Propagation Channel Modeling

The underwater acoustic channel is a time varying and space varying random channel of high environmental noise, a narrow channel bandwidth, large transmission loss and a serious multi-path effect. Because of the slow underwater movement of the AUV, the acoustic channel can be seen as a slow time-varying system. The system can be approximated as a LTI ( linear time-invariant )system. Suppose that there are n paths for the acoustic signal to transmit from the sound source to the hydrophones, and then, the unit impulse response of the multi-path channel from the sound source to the hydrophones will be:

(36) $$h(t)=\sum_{i=1}^{N}a_{i}\delta (t-\tau_{i})$$

**Figure 10:**
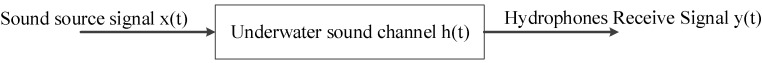
A simplified model of underwater sound transmission.

$a_{i}$ is the attenuation coefficient of the No. *i* transmission path; $\tau_{i}$ is the relative time delay of the transmission along the No. *i* transmission path. The underwater acoustic signal transmission can be simplified as the model that is shown in Figure 10:

Then, the hydrophones receive signal *y(t)* as a convolution of sound source signal *x(t)* and the unit impulse response *h(t)*, namely:

(37) y(t)=x(t)*h(t)

**Figure 11:**
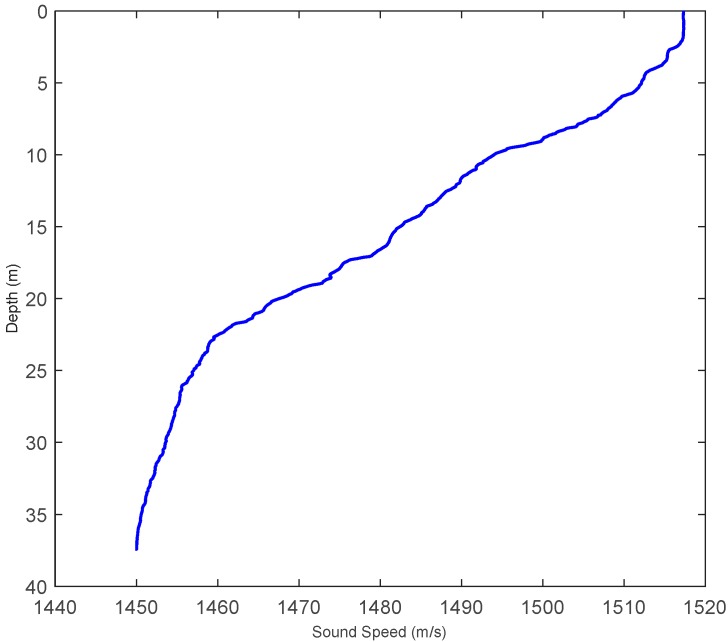
Underwater sound velocity distribution curve of a lake.

**Figure 12:**
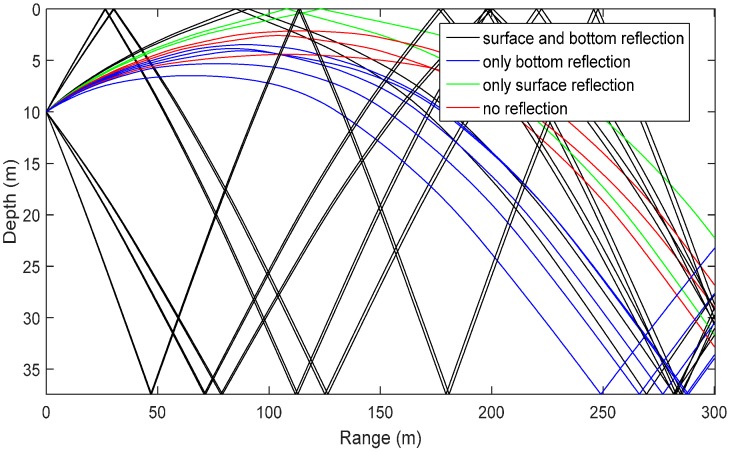
Sound ray transmission path.

Porter, M.B. *et al.* have developed BELLHOP software, which simulates the marine environment according to this model. This software can acquire amount N, the angle of incidence, the range and the time delay of intrinsic sound rays and provide the unit impulsive response of the system by inputting marine environment parameters [23]. This paper, according to the sound velocity distribution curve under a lake, which is as shown in Figure 11, adopts the BELLHOP software to establish an underwater sound signal transmission channel model, which will describe the sound field of the underwater environment and calculate the sound ray transmission path by setting the positions of the sound source and hydrophones, as shown in Figure 12; then, it will solve the unit impulse response function *h(t)* of the system and, finally, acquire the received signals of the hydrophones by the convolution operation and simulation.

**Figure 13:**
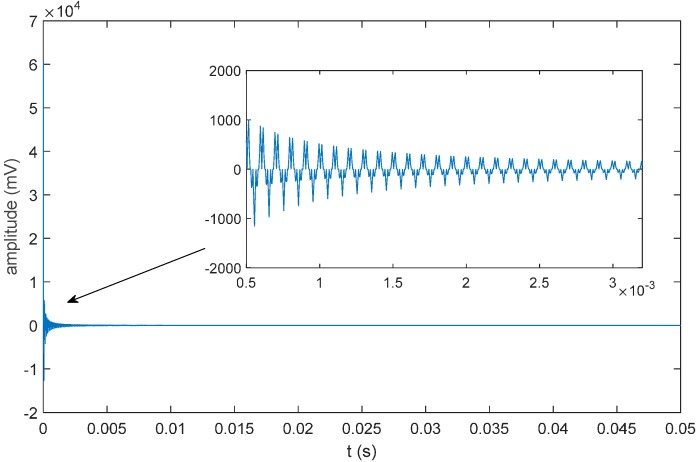
Oscillogram of the sound source signal.

The simulated sound source signal adopts the amplitude-modulated signal expressed in Equation (38). Its bandwidth is 50 kHz, and its center frequency is 25 kHz. Suppose that the noise model of the facility is a white noise model; then, the waveform of the sound source signal will be as shown in Figure 13.

(38) $$sigIN=(\sum_{i}^{N}a\sin(2\pi i\omega t))\cos(2\pi \omega_{c}t)$$

**Figure 14:**
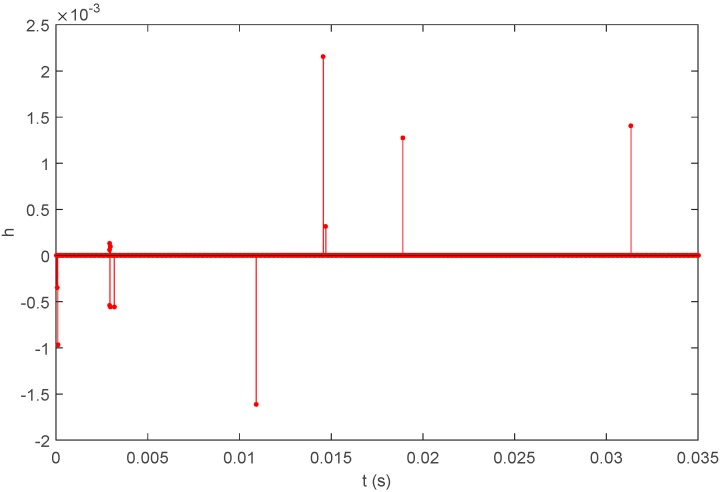
Unit impulse response h(t).

**Figure 15:**
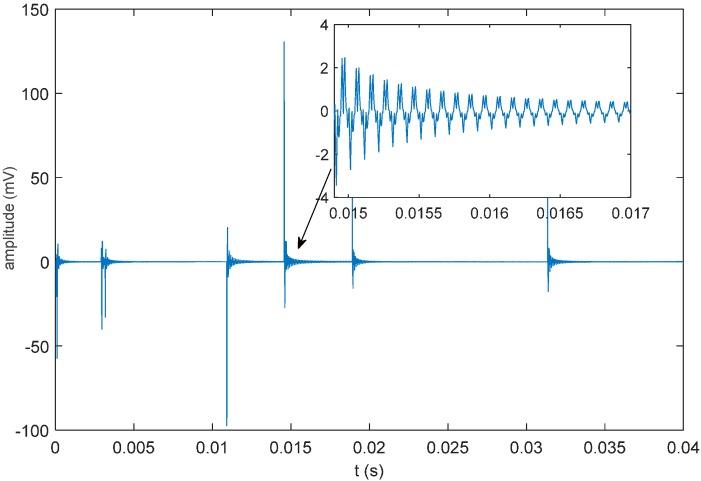
Oscillogram of received signals.

Calculate the unit impulse response as shown in Figure 14 according to the propagating sound rays, and the received signal can be calculated as shown in Figure 15 according to Equation (37). Adopt the sound field model established by BELLHOP and the underwater sound velocity distribution to simulate the situation of reflection and refraction, which may occur in the acoustic signal transmission process, as well as the signals received by the hydrophones, all of which will be used for the simulation and positioning calculation.

### 4.2. Simulation of SINS Assistance in the LBL Positioning Algorithm

**Figure 16:**
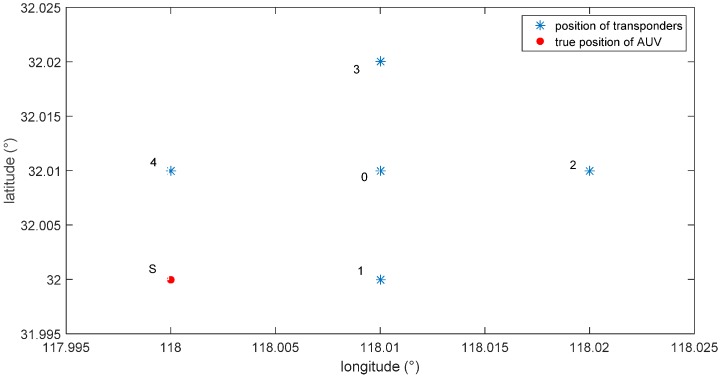
Layout of the hydrophones and the AUV.

As shown in Figure 16, set the positions of the hydrophones and the AUV; place five hydrophones underwater; their positions will be expressed with longitude and latitude, and they are respectively(118.01°, 32°), (118.01°, 32.01°), (118.01°, 32.02°), (118.02°, 32.01°), (118°, 32.01°); all of their depths are 30 m. Suppose that the current actual position of the AUV is (118°, 32°) with a depth of 10 m. The underwater acoustic velocity distribution data are acquired by an experiment on this lake. Simulate the acoustic signals received by the hydrophones according to the method introduced in Section 4.1, and calculate the time difference of different hydrophones receiving the signal.

#### 4.2.1. Simulation of SINS Assisting LBL in Tracking the Optimal Time Difference

**Figure 17:**
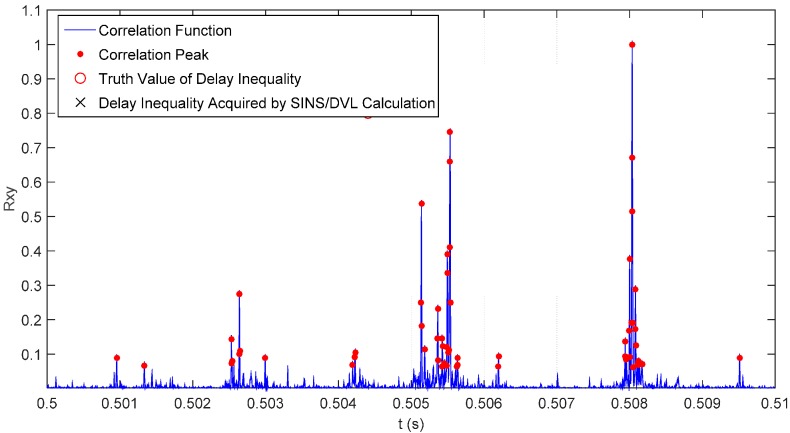
Screening out of the main correlation peak.

As shown in Figure 17, as the sound source signal will go through multiple paths, there will be also multiple correlation peaks in the generalized cross-correlation result. The correlation peaks whose amplitude is in the top twenty can be selected as alternative correlation peaks, while others are neglected due to excessive signal attenuation. The distance between the time difference with the highest amplitude (Point A in Figure 17) and the true time difference (the red circle in Figure 17) is relatively larger, so Point A cannot be selected as the true time difference. Using the proposed method, the time difference (symbol × in Figure 16), which is calculated through Equation (10) by the aiding of the SINS/DVL integrated navigation, better approaches the truth value. Therefore, the alternative correlation peak (Point B in Figure 17), which is closest to the symbol “×”, is selected as the ideal time difference, because it has the smallest distance to Point “A”. Traditional algorithms directly take the correlation peak that is corresponding to the maximum peak value as the main correlation peak to calculate the time difference; there will be larger errors under the multi-path effect. The improved algorithm adopted in this paper selects the main correlation peak through the positions obtained by the SINS/DVL integrated navigation system. A comparison of the calculation results and errors of the two algorithms is as shown in Table 1 and Table 2, which show that: the improved algorithm reduces the interference of multiple correlation peaks with time difference estimation under the multi-path effect and makes the calculation value accuracy of the final time difference superior to that of the traditional algorithm.

**Table 1:** Comparison of the calculation results of the time differences.

Time Difference of Two Hydrophones Receiving the Signal	Traditional Algorithm/s	Improved Algorithm/s	Truth Value/s
Ts1−Ts0	−0.3532	−0.3436	−0.3470
Ts2−Ts0	0.5080	0.5043	0.5044
Ts3−Ts0	0.6623	0.6499	0.6492
Ts4−Ts0	−0.2388	−0.2297	−0.2297

Note: The expression (Ts1−Ts0) represents the time difference from the sound source from the sound source propagating to Hydrophone 1 and Hydrophone 0, and so on.

**Table 2:** Error comparison of the calculation values of the time difference.

Time Difference of Two Hydrophones Receiving the Signal	Error of the Traditional Algorithm/s	Error of the Improved Algorithm/s
Ts1−Ts0	0.0062	0.0034
Ts2−Ts0	0.0036	0.0001
Ts3−Ts0	0.0132	0.0007
Ts4−Ts0	0.0091	0.0001

#### 4.2.2. Simulation of the Acoustic Velocity Correction Algorithm

The traditional algorithm directly adopts acoustic velocity in a traditional sense to calculate the distance difference. The improved algorithm adopted in this paper calculates equivalent acoustic velocity, which will be used to calculate the distance difference. A comparison of the calculation results and errors of the two algorithms is as shown in Table 3 and Table 4, which show that: as the improved algorithm adopts the equivalent acoustic velocity to calculate the distance difference, it corrects the sound velocity, under situations of the multi-path effect and sound ray curve. It greatly enhances the accuracy of the calculation values of the distance difference. Therefore, adopting the time difference algorithm and distance difference algorithm in this paper can greatly reduce the errors of the time difference and distance difference, which play a significant role in improving the AUV underwater positioning accuracy.

**Table 3:** Comparison of the calculation results of the distance difference.

Distance Difference of Two Hydrophones Receiving the Signal	Traditional Algorithm/m	Improved Algorithm/m	Ideal Distance Difference/m
Ds1−Ds0	−523.8619	−509.5792	−510.0840
Ds2−Ds0	739.6344	734.1894	734.3538
Ds3−Ds0	973.0620	954.7788	954.1334
Ds4−Ds0	−360.3024	−346.6323	−346.1796

Note: The expression (Ds1−Ds0) represents the distance difference from the sound source propagating to Hydrophone 1 and Hydrophone 0, and so on.

**Table 4:** Error comparison of the calculation values of the distance difference.

Distance Difference of Two Hydrophones Receiving the Signal	Error of the Traditional Algorithm/m	Error of the Improved Algorithm/m
Ds1−Ds0	13.7779	0.5048
Ds2−Ds0	5.2806	0.1644
Ds3−Ds0	18.9286	0.6454
Ds4−Ds0	14.1228	0.4527

#### 4.2.3. Simulation of the TDOA Positioning Algorithm

In order to verify the positioning effect of the algorithm, we conduct a simulation under the MATLAB environment. Adopt the BELLHOP model to simulate the acoustic signal receiving from the hydrophones, and calculate the time difference and distance difference; finally, adopt the position obtained from SINS/DVL integrated navigation system as the iterative initial value of the Taylor series expansion method to solve the positioning results; then obtain the longitude, latitude and depth of AUV, and make a comparison with the positioning results of the traditional algorithm. The result is as shown in Figure 17.

**Figure 18:**
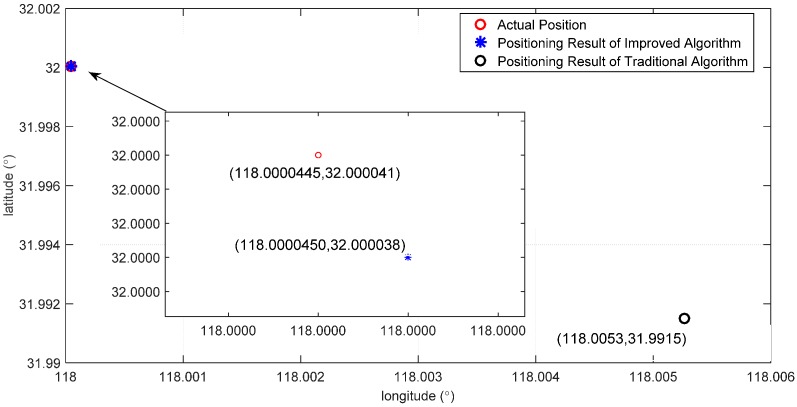
Comparison of the positioning results.

It can be seen from Figure 18 that: the positioning result of the traditional algorithm has far deviated from the actual position, while that of the improved algorithm is very proximate to the actual value. This is because the error of the distance difference of the traditional algorithm is larger, which results in the non-convergence of the positioning result or big error; then, an acceptable positioning result cannot be acquired. However, the error of the distance difference of the improved algorithm is less than 1 m, and a more accurate positioning result can be acquired finally.

### 4.3. Dynamic Simulation of AUV Integrated Navigation System Based on SINS/LBL/DVL/MCP

**Figure 19:**
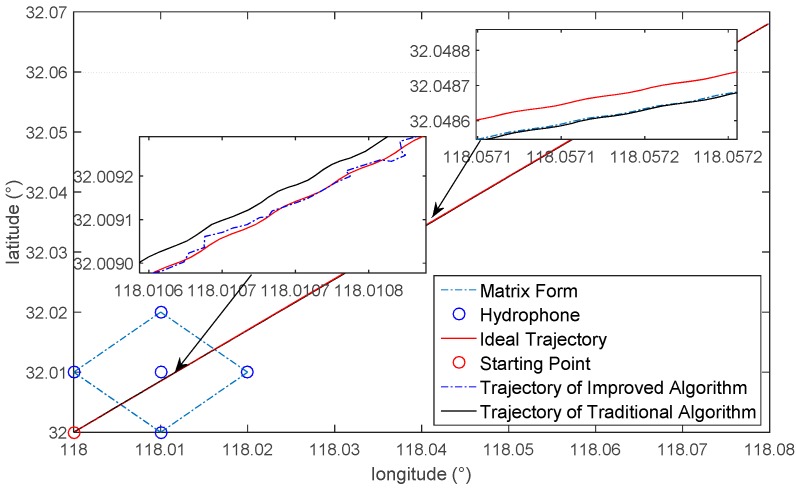
Dynamic simulation of the AUV integrated navigation system.

**Figure 20:**
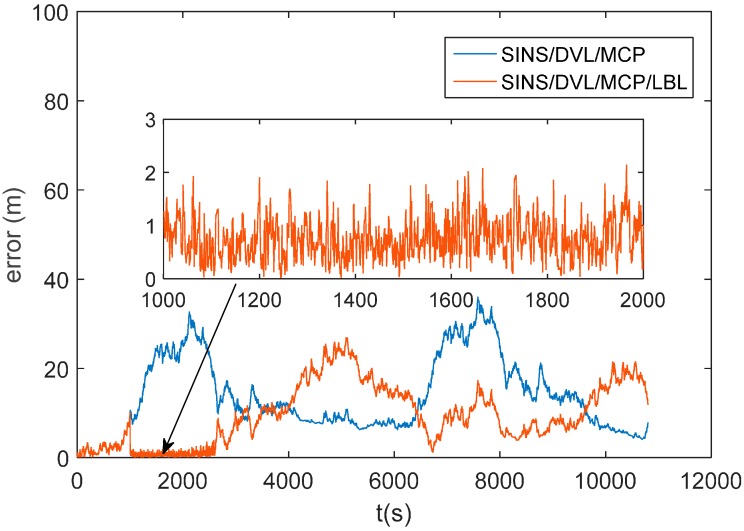
Error comparison of the positioning results.

In order to further verify the effectiveness of this algorithm when the AUV is in dynamic operation, a dynamic simulation has been performed on this algorithm. Place five hydrophones underwater; their positions are the same as the above simulation. Suppose that the AUV starts from (118°, 32°) and moves along north by east 45°, which is as shown in Figure 19. The random shift and constant value shift of the gyroscope is 50 μg; the constant value bias is 50 μg. The initial misalignment angles are respectively: pitching angle 1.5°, roll angle 1.5° and course angle 1.5°. The velocity error of DVL is 0.2 m/s. The heading error of MCP is 0.3°. The velocity of the AUV is 1 m/s. Adopt the algorithm in this paper to perform the positioning calculation, and integrate the positioning results with the SINS/DVL/MCP integrated system to calibrate accumulative errors; simulation time is three hours. In order to enhance the fault-tolerant ability of the system, if the positioning results are not converged, then the positioning results of LBL will not be used for the calibration. Figure 19 indicates that: when the AUV enters the scope of the hydrophones, the trajectory of the improved algorithm is fundamentally overlapping with the ideal trajectory, and that of the traditional algorithm deviates from the ideal trajectory. When the AUV leaves the scope of the hydrophones, the trajectory of both the traditional and improved algorithms deviates from the ideal trajectory. Figure 19 is a comparison graph of the positioning errors of the traditional and improved algorithms. The positioning errors will be expressed by the distance between the positioning results and the actual position. Figure 20 indicates that: within 0~1000 s, the positioning errors of both the traditional and improved algorithms will gradually enlarge, because within this period, the AUV is somewhat far away from the scope of the hydrophones; it takes a long time for the hydrophones to receive the sound source signal, and there will be great delay for the positioning results. Hence, LBL will not be used to calibrate within this period; instead, the SINS/DVL/MCP integrated navigation system will be used to perform the positioning. When the AUV approaches the scope of hydrophones, namely after 1000 s, we adopt LBL to perform the calibration. As the errors of adopting the traditional algorithm to calculate are larger, the LBL positioning results will not converge, which has nearly no contribution to calibrating for the SINS/DVL/MCP integrated navigation system. Within 1000 s to 3000 s, the positioning errors enlarge gradually, with the maximum reaching 30 m or so. However, the improved algorithm in this paper greatly reduces the errors of the calculation values of the distance difference. The LBL positioning results can calibrate the accumulative errors of the integrated navigation system, which will control the positioning errors within 2 m. When the AUV leaves the scope of the hydrophones, the LBL positioning system will lose its effect; however, in general, the precision is superior to the system that has not been calibrated by LBL. The above analysis shows that: under actual dynamic operation, when the AUV approaches the scope of the hydrophones, the improved algorithm can be used to perform the positioning calculation, and the calculation results can be used to calibrate the SINS/DVL/MCP integrated navigation system, which will greatly improve the positioning accuracy of the AUV when it navigates underwater.

## 5. Conclusions

This paper, directed at deficiencies in existing underwater positioning technology, proposes an underwater positioning system based on the mutual assistance of SINS/DVL/MCP and LBL. The whole system consists of an SINS/DVL/MCP integrated navigation system and an LBL underwater acoustic positioning system. The latter adopts the TDOA positioning algorithm based on Taylor series expansion, as well as the positioning results provided by the SINS/DVL/MCP integrated navigation system, which will assist in calculating the delay inequality and distance difference. In the meantime, the positioning results will also be used as the iterative initial value of the positioning algorithm. The final positioning results will be used to calibrate the accumulative errors of the position of the SINS/DVL/MCP integrated navigation system.

The final simulation results show that: compared to the traditional algorithm, this scheme can not only directly correct accumulative errors caused by the dead reckoning algorithm, but also solves the problem of ambiguous correlation peaks caused by the multipath transmission of underwater acoustic signals. This algorithm can greatly improve the AUV underwater positioning accuracy. Only when the AUV installed with the SINS/DVL integrated navigation system approaches the scope of the hydrophones can accumulative errors be effectively calibrated. Hence, it has strong practicability.
